# The role of PCR in the diagnosis of tuberculous meningoencephalitis in children – Case report

**DOI:** 10.1186/1471-2334-14-S7-P71

**Published:** 2014-10-15

**Authors:** Mădălina Merişescu, Adrian Streinu-Cercel, Dragoş Florea, Gheorghiță Jugulete, Endis Osman, Anca Drăgănescu, Angelica Vişan, Anuța Bilaşco, Monica Luminos

**Affiliations:** 1 National Institute for Infectious Diseases "Prof. Dr. Matei Balş", Bucharest, Romania; 2 Carol Davila University of Medicine and Pharmacy, Bucharest, Romania

## Background

Tuberculous (TB) meningoencephalitis is an extremely severe condition which requires a quick and correct diagnosis in order to institute a specific etiological treatment in a timely manner. The current “classical” diagnosis of TB meningitis is established through CSF cultures. The result is often inconclusive or is heavily delayed in the case of positive cultures (4-6 weeks). PLEX-ID is a new method which can establish an etiological diagnosis of bacterial infection in a matter of hours by detecting bacterial DNA in various pathological products (blood, CSF, synovial and pleural fluid).

## Case report

We present two cases admitted in the Pediatric Intensive Care Unit of the National Institute for Infectious Diseases "Prof. Dr. Matei Balş" with the suspicion of acute TB meningoencephalitis. The first case was a boy of 4 years old, and the second is represented by a little girl aged 4 years and 6 months. The positive diagnosis was established though classic clinical and laboratory criteria and confirmed by molecular methods of diagnosis (bacterial PCR). The treatment was given immediately after admission in the first case, but the second child was transferred to our clinic in severe condition, with external ventricular shunt for hydrocephalus and she started the treatment after 3 weeks from onset.

We have monitored the correlation of the data obtained through classical molecular methods as well as the clinical and biological evolution of the patients under anti-tuberculous treatment. Etiology was determined by bacterial PCR obtained from CSF which identified *Mycobacterium tuberculosis* as the causative agent responsible in both cases. Evolution was different in these cases. In the first case, because treatment was given in the first hours after admission, the evolution was favorable, but in the second one unfortunately it was fatal.

## Conclusion

TB meningoencephalitis represents a severe condition with a high mortality rate and high frequency of neurological sequelae, which imposes urgent institution of adequate treatment. New molecular methods are very helpful both in establishing an early diagnosis and in determining antibiotic sensitivity to best choose a correct and efficient therapeutic regimen.

## Consent

Written informed consent was obtained from the parents for publication of this Case report and any accompanying images. A copy of the written consent is available for review by the Editor of this journal.

